# Combination of Immune-Related Genomic Alterations Reveals Immune Characterization and Prediction of Different Prognostic Risks in Ovarian Cancer

**DOI:** 10.3389/fcell.2021.653357

**Published:** 2021-04-23

**Authors:** Xibo Zhao, Shanshan Cong, Qiuyan Guo, Yan Cheng, Tian Liang, Jing Wang, Guangmei Zhang

**Affiliations:** Department of Gynecology, The First Affiliated Hospital, Harbin Medical University, Harbin, China

**Keywords:** ovarian cancer, TCGA, LASSO, prognosis, risk score model, immune genes

## Abstract

With the highest case-fatality rate among women, the molecular pathological alterations of ovarian cancer (OV) are complex, depending on the diversity of genomic alterations. Increasing evidence supports that immune infiltration in tumors is associated with prognosis. Therefore, we aim to assess infiltration in OV using multiple methods to capture genomic signatures regulating immune events to identify reliable predictions of different outcomes. A dataset of 309 ovarian serous cystadenocarcinoma patients with overall survival >90 days from The Cancer Genome Atlas (TCGA) was analyzed. Multiple estimations and clustering methods identified and verified two immune clusters with component differences. Functional analyses pointed out immune-related alterations underlying internal genomic variables potentially. After extracting immune genes from a public database, the LASSO Cox regression model with 10-fold cross-validation was used for selecting genes associated with overall survival rate significantly, and a risk score model was then constructed. Kaplan–Meier survival and Cox regression analyses among cohorts were performed systematically to evaluate prognostic efficiency among the risk score model and other clinical pathological parameters, establishing a predictive ability independently. Furthermore, this risk score model was compared among identified signatures in previous studies and applied to two external cohorts, showing better prediction performance and generalization ability, and also validated as robust in association with immune cell infiltration in bulk tissues. Besides, a transcription factor regulation network suggested upper regulatory mechanisms in OV. Our immune risk score model may provide gyneco-oncologists with predictive values for the prognosis and treatment management of patients with OV.

## Introduction

Ovarian cancer (OV) is the second leading cause of gynecological cancer and has the highest case-fatality rate among women, with 21,750 new cases and 13,940 deaths predicted for 2020 in the United States (Siegel et al., 2020). About 90% of patients suffering from OV have epithelial OV, which means it is of epithelial origin. High-grade serous ovarian cancer is the most common histological and the most aggressive subtype (Gloss and Samimi, 2014), with almost 80% of patients diagnosed as late stage and an approximately low 5-year survival rate of 30–40% due to concealing without effective characteristics (Dao et al., 2016; Torre et al., 2018). Despite the continuous progress in its diagnosis and treatment, the low sensitivity or specificity of the common OV biomarkers used for clinical diagnosis and recurrence surveillance, as well as the standard treatment that has no advanced improvement beyond cytoreductive surgery and platinum-based combination chemotherapy in decades, still makes it a significant threat to women’s lives. Therefore, it is of importance to understand the mechanisms of OV through its development and progression.

The development of OV is complex with several histopathological types and involves multiple alterations of oncogenes and tumor suppressor genes. Great efforts have been made to identify potential genomic alterations, either individually or jointly, many of which have been validated as major risk indicators for mortality; for instance, ERBB2 had been demonstrated as a poor prognostic predictor with elevated expression, and the combined expression of MANF and DOCK11 was identified as a novel risk factor (Qiu et al., 2014; Luo et al., 2018; Liu et al., 2020a; Tang et al., 2020). However, the functions of non-cancer cells such as stromal or immune cells and non-cellular components in a tumor microenvironment (TME) and their interactions are still poorly understood even though plenty of studies and clinical trials have been conducted for the purpose of improved survival rate and reduced chemotherapy resistance. Moreover, the TME has increasingly been shown to manipulate aberrant histological and cellular functions and plays a critical role in the subsequent evolution of malignancies, more progressive and resistant to chemotherapy (Mroue and Bissell, 2013). Accumulating evidence is uncovering the crucial roles of immunity in tumor immunosurveillance (Dunn et al., 2004; Koebel et al., 2007; Finn, 2008). Other studies of the TME during tumor development reveal multi-omics prognostic biomarkers that may be used for imaging or liquid biopsy analysis, both important to select the most suitable therapies and stratification of patients, including OV (Abadjian et al., 2017; Wu et al., 2017; Willumsen et al., 2018; Guo et al., 2019; Jiang et al., 2020). However, because of heterogeneity and developing drug resistance, consistent with low mutational burden, patients with OV often show a lower response to immunotherapy (Zhu et al., 2018). The lack of successful treatment leads us to measure comprehensive genomic and epigenomic alterations that affect outcomes and constitute therapeutic targets, and thus, further research studies are still needed urgently.

In this study, we employed high-throughput gene expression profiles with complete clinical pathological information offered in public databases to identify genes and features involved in immune-related processes and the prognosis of OV. Multiple machine-learning-based methods were employed to investigate and validate relative immune components and their interactions. An immune gene-based risk score model was constructed and verified using available clinical data. In summary, our findings may provide new ideas and targets for the precious medication of OV.

## Materials and Methods

### Data Collection and Processing

The fragments per kilobase million (FPKM) expression profile of TCGA RNA-sequencing data (level 3) for OV and the corresponding clinical pathological parameters were downloaded from UCSC Xena genome browser^1^ (Goldman et al., 2020). Also, annotation information mapping probes to gene symbols was obtained from the GENCODE database^2^, using the version for human release 22 (Frankish et al., 2019). To normalize both gene size and library size, FPKM values were then transformed to transcripts per million (TPM) (Wagner et al., 2012). Duplicated genes with the same stable ensemble ID were merged by their average values. Clinical data with paired expression data were then abstracted and summarized by the following criteria: (i) duplicated samples with both formalin-fixed and paraffin-embedded and frozen tissues subjected to sequencing analysis were removed, retaining one; (ii) patients without well-annotated clinical information were removed; and (iii) patients with overall survival time <90 days were also removed from the current research. The whole cohort was then stratified for training and testing the risk models using the methods below.

Two additional datasets – GSE9891 and GSE14764 – were downloaded using ‘‘GEOquery’’ package from the Gene Expression Omnibus (GEO) for external validation^3^. Additionally, a comprehensive immune gene dataset was obtained from the ImmPort database^4^ to filter genes enrolling in immune or inflammatory response for a more specific inspection (Bhattacharya et al., 2018).

### Investigation of Tumor Infiltration Lymphocyte Subpopulations, Dimensionality Reduction, and Cluster Analysis

In the current analysis, the single-sample gene set enrichment analysis (ssGSEA) algorithm was employed to comprehensively identify immune cell types that are overrepresented in the TME calculating individual enrichment score (ES) based on weighted difference of the empirical cumulative distribution for each pairing of a sample and gene set (Barbie et al., 2009). Immune marker gene panels were collected from a literature resource representative of 28 subpopulations of tumor infiltration lymphocytes (TILs) related to both innate and adaptive immunity, and these genes are expressed neither in cancer nor normal tissues (Charoentong et al., 2017). These TILs were further classified into three categories based on their functional orientations. The enrichment score was then normalized by the min–max algorithm:

$$z=\frac{x-\text{min}\left(x\right)}{\max\left(x\right)-\text{min}\left(x\right)}$$

Here, *x* is the enrichment score calculated by ssGSEA.

Unsupervised hierarchical clustering was then performed on the basis of the Euclidean distance and complete linkage provided in “dist” and “hclust” functions, respectively, dividing OV patients into “Immune High” and “Immune Low” clusters for exploration of differentially expressed transcription patterns between these two clusters. t-Distributed stochastic neighbor embedding (t-SNE) is a popular nonlinear dimensionality reduction technique achieved *via* t-SNE modeling the probabilities as a Gaussian distribution computing low-dimensional coordinates of high-dimensional data embedding to a dimensionally reduced space using Cauchy distribution (Student’s *t*-distribution with 1 degree of freedom), often called a map (Belkina et al., 2019). These two clusters further confirmed the robustness using the “Rtsne” package.

### Identification of Immune-Associated Components and Tumor Purity

We obtained the three scores of Estimation of STromal and Immune cells in MAlignant Tumor tissues using Expression data (ESTIMATE) described before to calculate the stromal and immune scores that represent the infiltration of stroma or immune cells in tumor tissues, as well as estimate scores, from which tumor purity can be inferred (Yoshihara et al., 2013). The algorithm was implemented based on “estimate” R package, and the Wilcoxon signed-rank test was used for comparisons between the two clusters.

The cell type identification by estimating relative subsets of RNA transcripts (CIBERSORT) uses a set of reference gene expression termed leukocyte gene signature matrix (LM22) containing 547 genes to normalize gene expression profiles, and quantifies either relative or absolute cell components with a support vector machine (Newman et al., 2015). An inference of 22 types of immune cell matrix following pairwise Pearson’s correlation coefficients and root mean square errors (RMSE) and empirical *p* values were also obtained at 1,000 permutations. These further determined the immune heterogeneity in different immune clusters and the correlation between genomic alterations and LM22.

### Exploration of Differentially Expressed Patterns and Functional Enrichment Analysis

The “limma” package was used to screen out the differentially expressed genes (DEGs) between clusters. Gene Ontology (GO) annotation and Kyoto Encyclopedia of Genes and Genomes (KEGG) enrichment analyses were carried out using the “clusterProfiler” package to obtain classified annotations and functional enrichment of DEGs (Yu et al., 2012). Additionally, gene set enrichment analysis for biological pathways was conducted by GSEA, a Java-based software (Subramanian et al., 2005). The annotated gene sets were specified by “hallmarker gene sets” and “c7 immunologic signature gene sets” downloaded from MSigDB as input files (Liberzon et al., 2015; Godec et al., 2016). The significance for the corresponding enriched terms was statistically set as *p* < 0.05 adjusted by the Benjamini–Hochberg (BH) method and visualized using the “enrichplot” package.

### Least Absolute Shrinkage and Selection Operator Cox Regression

Based on the intersection of DEGs and genes offered in the ImmPort database, we explored the potential interactions among immune genes indicating prognosis. Least absolute shrinkage and selection operator (LASSO) Cox regression provided in “glmnet” package was used for a linear combination, performing continuous shrinkage and also feature selection (Tibshirani, 1997). Currently, LASSO is widely used for the survival analysis of high-dimensional data (Jiang et al., 2018). Ten-fold cross-validation was used in this study to derive the best-fit lambda value while minimizing the mean cross-validated error. A LASSO Cox model was constructed through the formula:

$$\mathrm{Risk}\mathrm{score}=\sum_{i=1}^{n}\text{coefi}*\text{expi}$$

Here, *n* represents the number of selected prognostic genes, and coef*_*i*_* is the coefficient of each non-zero gene*_*i*_*, while exp*_*i*_* represents the expression value of each screened gene*_*i*_* contributing to the model. Each sample enrolled in the study was then calculated using the formula and grouped for subsequent analyses.

### Survival Analysis and Model Judgment

Analyses were performed in each cohort independently. The patients in the two clusters and the whole TCGA cohort were then categorized into high group and low group, respectively, after calculating the optimal cutoff value provided in “surv_cutpoint” function based on the risk score model. The overall survival (OS) rates between each of the two subgroups were compared using Kaplan–Meier survival curves, and statistical significance was implemented by the log-rank test. Patients with censored values were marked as “+,” and their survival curves were also plotted. The univariate and multivariate Cox regression analyses were also implemented among clinical pathological variables, and the risk score model was also implemented using “ezcox” package (Wang et al., 2019). Finally, time-dependent receiver operating characteristic (ROC) curve analysis was performed to assess OS prediction of sensitivity and specificity. Additionally, patients’ risk scores and survival status, as well as expressed patterns of identified prognostic genes, were also explored to illustrate their distributions with different stratifications of clinical parameters.

Meanwhile, two external cohorts from GEO were employed to validate our risk score model. Samples only with the same pathological diagnoses consistent with the TCGA cohort remained, consisting of 251 and 68 samples, respectively. Besides, our risk score model was compared among several prognostic biomarkers identified previously, revealing stability and reliability in predicting OS.

### Statistical Analysis

Statistical analyses were all performed on R software version 3.6.3^5^. Comparisons between two variables were performed by the Wilcoxon signed-rank sum test. For comparisons of more than two variables, the Kruskal–Wallis test was performed. Hazard ratio (HR) and 95% confidence intervals (CI) for each variable were also calculated where needed. A two-sided, *p* value < 0.05 adjusted by the BH or false discovery rate (FDR) method and | log2-fold change (FC)| > 1 were regarded as statistically significant thresholds.

## Results

### Summary of Expression and Clinical Pathological Data

After obtaining the expression of all probes as well as clinical pathological variables from the UCSC Xena database, we set up the criteria for more rigorous strength, as samples with OS < 90 days excluded meant more evidence about treatment and medication. Here, a total of 309 samples expressing 19,711 mRNAs were enrolled. An overview of the patients included in the whole TCGA cohort and of each cluster is shown in Table 1.

**TABLE 1:** Clinical pathological characteristics of OV patients in TCGA (*n* = 309).

Characteristics	Number of patients			*p*	*p* adjusted
	Overall	Immune High	Immune Low		
	*n* = 309	*n* = 194	*n* = 115		
Age				0.46	0.6975
Mean (SD)	59.28 (11.66)	58.91 (11.60)	59.92 (11.76)		
Range	30–87	30–87	34–87		
Age group				0.607	0.725625
≤58 (%)	155 (50.2)	100 (51.5)	55 (47.8)		
>58 (%)	154 (49.8)	94 (48.5)	60 (52.2)		
Stage				0.398	0.6975
II (%)	18 (5.8)	14 (7.2)	4 (3.5)		
III (%)	252 (81.6)	156 (80.4)	96 (83.5)		
IV (%)	39 (12.6)	24 (12.4)	15 (13.0)		
Grade				0.645^a^	0.725625^a^
G1 (%)	1 (0.3)	1 (0.5)	0 (0.0)		
G2 (%)	36 (11.7)	22 (11.3)	14 (12.2)		
G3 (%)	271 (87.7)	171 (88.1)	100 (87.0)		
G4 (%)	1 (0.3)	0 (0.0)	1 (0.9)		
Lymphatic invasion				0.27	0.6975
No (%)	37 (32.2)	21 (28.0)	16 (40.0)		
Yes (%)	78 (67.8)	54 (72.0)	24 (60.0)		
Tumor residual disease				0.465	0.6935
No (%)	58 (20.9)	31 (17.9)	27 (25.7)		
1–10 mm (%)	142 (51.1)	93 (53.8)	49 (46.7)		
11–20 mm (%)	19 (6.8)	12 (6.9)	7 (6.7)		
>20 mm (%)	59 (21.2)	37 (21.4)	22 (21.0)		
Venous invasion				0.043	0.1935
No (%)	34 (40.5)	17 (31.5)	17 (56.7)		
Yes (%)	50 (59.5)	37 (68.5)	13 (43.3)		
Survival time (days)				0.891	0.891
Mean (SD)	1,200.22 (956.09)	1,194.45 (947.56)	1,209.97 (974.41)		
Range	90–5,481	90–5,481	92–4,624		
Survival status				0.026	0.1935
Alive (%)	134 (43.4)	94 (48.5)	40 (34.8)		
Dead (%)	175 (56.6)	100 (51.5)	75 (65.2)		

*^a^p and adjusted p values were determined using Fisher’s exact test when appropriate.*

### Construction of Immune-Related Clusters and Exploration Heterogeneity of Components

**FIGURE 1:**
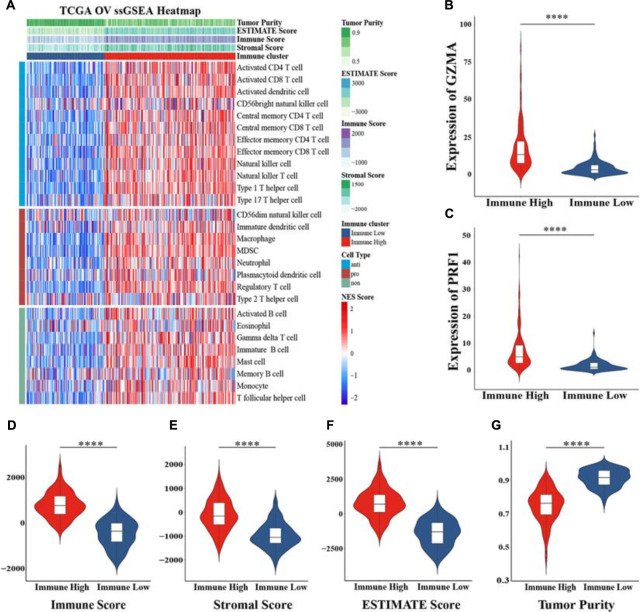
Construction of immune-related clusters and exploration heterogeneity of components. **(A)** Clusters were constructed in The Cancer Genome Atlas (TCGA) cohort determined by ssGSEA and unsupervised hierarchical clustering analysis, and heterogeneity was explored by the ESTIMATE algorithm. Violin plots showed the immune cytolytic activity between the expression of GZMA **(B)** and PRF1 **(C)** between two clusters. Distribution of immune score **(D)**, stromal score **(E)**, ESTIMATE score **(F)**, and tumor purity **(G)** in the “Immune High” cluster and the “Immune Low” cluster indicated heterogeneity between two clusters. NES, normalized enrichment score; ****p < 0.0001.

Using the ssGSEA method, we estimated 28 subpopulations of TILs including major types that participated in antitumor and promoted tumor procession closely linked with adaptive immunity and innate immunity functions or pathways, some of which are vital components of the tumor tissue. The whole TCGA cohort was split into two different clusters based on their normalized ES (NES) and unsupervised clustering analysis: “Immune High” (*n* = 194, 62.78%) and “Immune Low” (*n* = 115, 37.22%) (Figure 1A). Furthermore, we explored the expression of GZMA and PRF1, whose geometric mean value represents immune infiltration and immune cytolytic activity (Rooney et al., 2015). These two genes both showed higher expression in the “Immune High” cluster (Figures 1B,C). We also applied another unsupervised dimensionality reduction algorithm t-SNE confirming that two clusters possessed robust assignments, in accordance with former results (Supplementary Figures 1A,B). To further explore tumor purity and heterogeneity of components between two clusters, three scores according to the ESTIMATE algorithm were assessed. We found that immune scores, stromal scores, and estimate scores in the “Immune High” cluster were all significantly increased when compared with those in the “Immune Low” cluster, meaning higher infiltrations of immune and stromal cells in the “Immune High” cluster (Wilcoxon signed-rank test, *p* < 0.0001) (Figures 1D–F). However, tumor purity inferred from these three scores showed a significantly opposite trend between two clusters (Wilcoxon signed-rank test, *p* < 0.0001), indicating declined components of tumor cells comprised as integrated TME with non-tumor cells (Figure 1G). These results suggested the presence of intratumoral heterogeneity in OV, and stratification was observed even compared with different methods.

### Differentially Expressed Genes and Potential Mechanisms Underlying Immune-Related Roles

**FIGURE 2:**
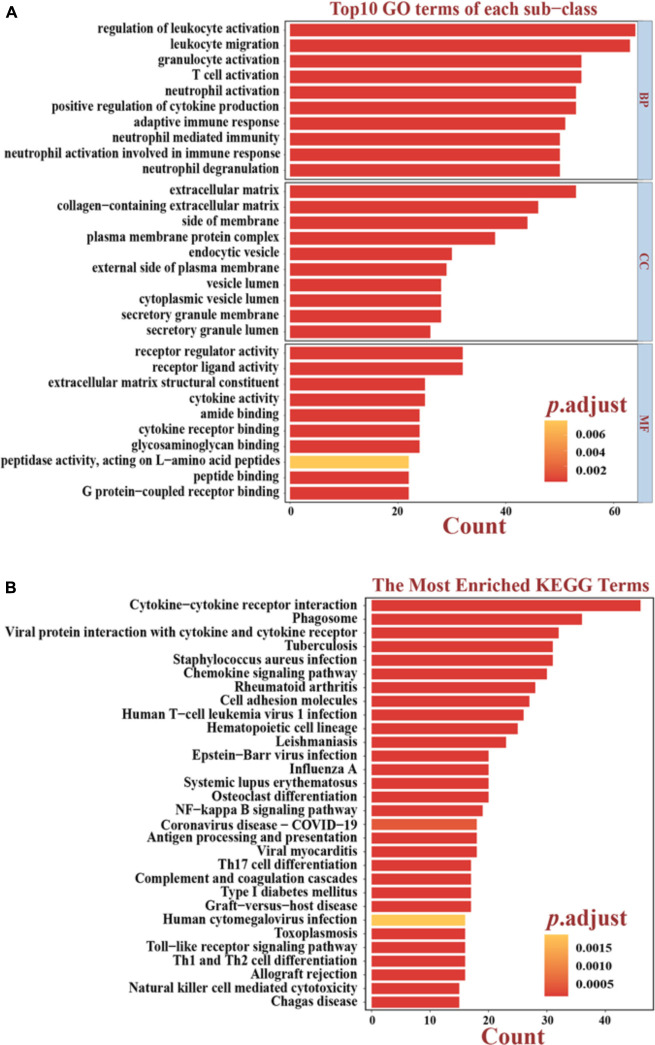
Functional analyses based on differentially expressed genes (DEGs). **(A)** Top 10 results of the GO enrichment analysis in BP, CC, and MF. The bright yellow-to-salmon pink chromatograms indicate the corresponding *p* values corrected by the BH method. **(B)** Top 30 results of the KEGG enrichment analyses. The bright yellow-to-salmon pink chromatograms indicate the corresponding *p* values corrected by the BH method. GO, Gene Ontology; KEGG, Kyoto Encyclopedia of Genes and Genomes; BP, biological processes; CC, cell components; MF, molecular functions.

**FIGURE 3:**
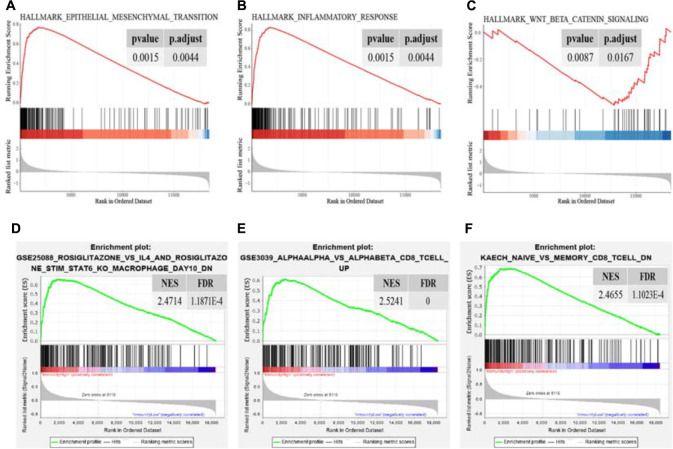
Significant enrichment terms for GSEA in “hallmarker gene sets” **(A–C)** and in “c7 immunologic signature gene sets” **(D–F)**. The significance of NES was all adjusted on behalf of the false discovery rate. NES, normalized enrichment score; FDR, false discovery rate.

To identify DEGs between the “Immune High” cluster and the “Immune Low” cluster, differential expression analysis was conducted, revealing and identifying a total of 381 DEGs, all of which were explored for functional analyses. The top 10 of the enriched GO terms significantly shown in the bar plot indicated that most of them were associated with immunological and tumor-associated processes, such as regulation of leukocyte activation, leukocyte migration, granulocyte activation, T-cell activation, and neutrophil activation in biological process (BP); receptor regulator activity, receptor-ligand activity, cytokine activity, and cytokine receptor binding in molecular function (MF); and extracellular matrix, plasma membrane protein complex, cytoplasmic vesicle lumen, and secretory granule membrane in cellular component (CC) (Figure 2A). The top 30 KEGG pathways enriched significantly also indicated immune-related terms such as cytokine–cytokine receptor interaction, phagosome, chemokine signaling pathway, cell adhesion molecules, NF-kappa B signaling pathway, Th17 cell differentiation, Th1 and Th2 cell differentiation and, unexpectedly, coronavirus disease 2019 (COVID-19) (Figure 2B). Recent studies have indicated that female cancer patients have an increased infection risk and develop more severe forms of COVID-19, and overexpression of CTSL pivotal for COVID-19 infection is a marker of invasion and metastasis in ovarian cancer (Sui et al., 2016; Liang et al., 2020; Rugge et al., 2020; Montopoli et al., 2021). These results may suggest a variety of coping strategies during a pandemic between inflammation and tumorigenesis. We also downloaded “hallmarker gene sets” and “c7 immunologic signature gene sets” as background gene sets for GSEA. These results indicated that immune signatures, such as “EPITHELIAL MESENCHYMAL TRANSITION” (*p* adjusted = 0.0044) and “INFLAMMATORY RESPONSE” (*p* adjusted = 0.0044), were most significantly enriched in patients in the “Immune High” cluster (Figures 3A,B and Supplementary Figures 2A–F). Additionally, a significant enrichment in the “Immune Low” cluster was “WNT_BETA_CATENIN_SIGNALING” (*p* adjusted = 0.0167) (Figure 3C). As for immunologic gene sets, we also found a positive enrichment in the “Immune High” cluster corresponding to immune cells and other relative terms (all *p* adjusted < 0.05) (Figures 3D–F and Supplementary Tables 1, 2). Importantly, these intimate relationships between clusters and immune-related gene sets were confirmed without a doubt. It could be suggested that exploration of pathways and signatures aforementioned in OV development and revealing inherent molecular mechanisms involved may be urgent.

### Extraction of Differentially Expressed Immune Genes and Construction of a Prognostic Model

**FIGURE 4:**
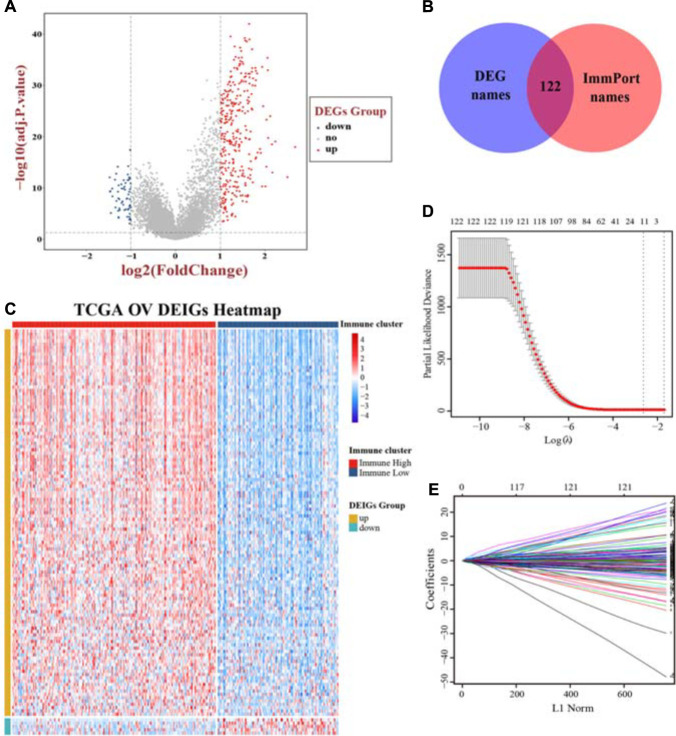
Screening immune-related genes and construction of a prognostic model. **(A)** A volcano plot of all DEGs was shown combined with | log2-FC| and adjusted *p* value. **(B)** Extraction of immune-related genes from DEGs. **(C)** The landscape of expression pattern for all DEIGs between two immune clusters. Ten-fold cross-validation for tuning parameter selection **(D)** and coefficients extraction **(E)** in the LASSO Cox model. DEGs, differentially expressed genes; DEIGs, differentially expressed immune genes.

Focusing on functional analyses, we next intended to extract immune-related genes from DEGs, including 322 upregulated genes and 59 downregulated genes (Figure 4A and Supplementary Figure 3A). Based on the ImmPort database, 122 genes were extracted and their expression patterns were explored as differentially expressed immune genes (DEIGs), consisting of 117 upregulated and 5 downregulated genes, respectively (Figures 4B,C). To acquire genes with the greatest potential prognostic values, we used the “Immune High” cluster as the training set. LASSO Cox regression analysis was performed with 10-fold cross-validation to evaluate and eliminate variables which contributed less to the model. Finally, a total of 11 mRNAs, namely MSR1, FPR1, RNASE2, GBP2, CXCL9, CXCL11, C5AR1, CCL13, FGF17, CXCL14, and PI3, related to OS with non-zero coefficients were selected as candidate predictors contributing to a linear model, and then they were validated and assessed, and significant differences in OS were observed (Figures 4D,E; Supplementary Figure 3B; Supplementary Table 3). The expression levels and regression coefficients were integrated, and therefore, a risk score model was developed. For further survival analyses, a risk score based on the model was calculated for each sample.

### Stratification of Samples and Verification of Independent Prognostic Model

**FIGURE 5:**
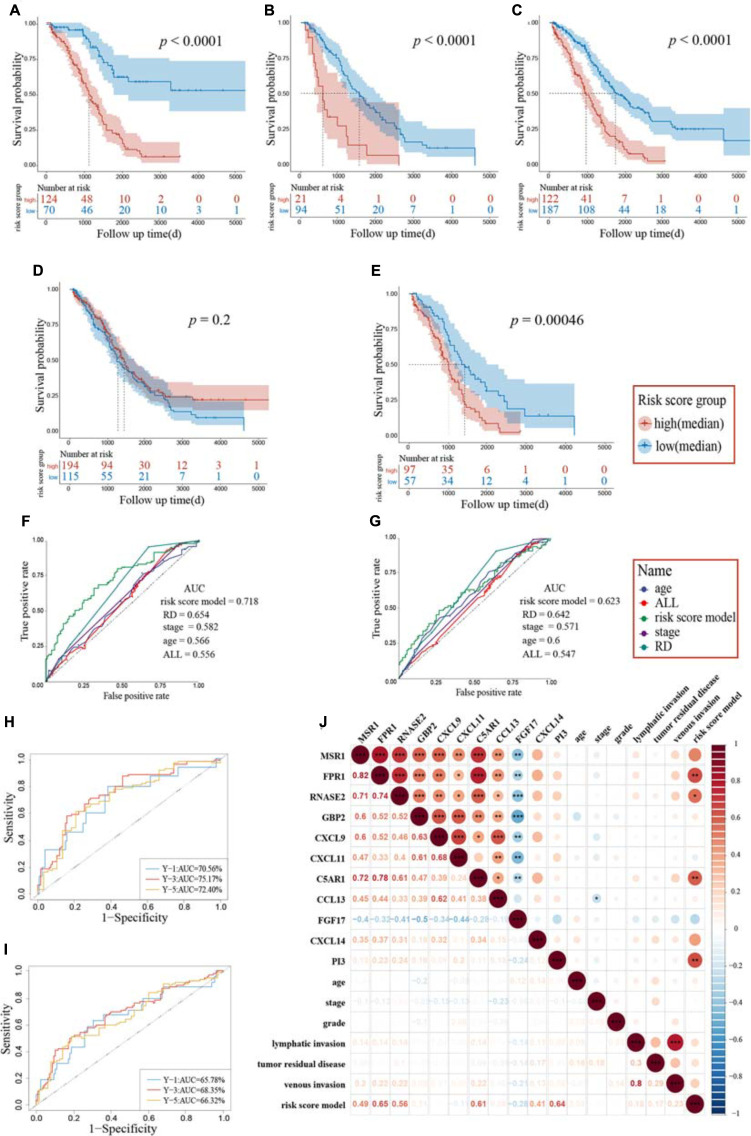
Assessment of candidate immune genes and prognostic capacity. A log-rank test was set up for statistics. Kaplan–Meier survival analysis based on the risk score model in the “Immune High” cluster **(A)**, the “Immune Low” cluster **(B)**, and the whole TCGA cohort **(C)**. Patients grouped by best cutoff values were listed in the risk score chart below at different time points. **(D)** Kaplan–Meier survival analysis between two immune clusters. **(E)** Kaplan–Meier survival analysis based on the risk score model between two immune clusters. Patients were grouped by median value in each cluster. ROC and the corresponding AUC of the risk score model and other clinical pathological characteristics for the “Immune High” cluster **(F)** and the whole TCGA cohort **(G)**. Time–ROC of the risk score model for the “Immune High” cluster **(H)** and the whole TCGA cohort **(I)**. The ROC and AUC of the predictions for 1, 3, and 5 years are shown, respectively. **(J)** Correlation heatmap of immune genes extracted by LASSO, risk score model, and clinical pathological characteristics. The lower triangular was the correlation coefficients between two variables and the upper triangular matrix was the significant adjusted *p* values. RD, tumor residual disease; ROC, receiver operating characteristic; AUC, area under the curve; *p < 0.05, **p < 0.01, ***p < 0.001.

The whole TCGA cohort and the “Immune Low” cluster were all enrolled for validation, and these three cohorts were stratified into high-risk group and low-risk group followed by the cutoff point. The Kaplan–Meier plot showed significant differences in terms of patients’ OS between the high- and low-risk groups: high-risk group (*n* = 124) versus low-risk group (*n* = 70) in the “Immune High” cluster (log-rank test, *p* < 1.0E-4) (Figure 5A); high-risk group (*n* = 21) versus low-risk group (*n* = 94) in the “Immune Low” cluster (log-rank test, *p* < 1.0E-4) (Figure 5B); and high-risk group (*n* = 122) versus low-risk group (*n* = 187) in the whole TCGA cohort (log-rank test, *p* < 1.0E-4) (Figure 5C). Though no significant difference in patients’ OS between two immune clusters was observed (log-rank test, *p* = 0.2, Figure 5D), we could also distinguish the significance stratified by the median cutoff point of each cluster, meaning that a higher risk score may more likely belong to the “Immune High” cluster and may predict a worse prognosis (log-rank test, *p* = 4.6E-5, Figure 5E).

Univariate Cox regression analysis was performed for these studies. The risk score model was independently a negative prognostic factor for the training cohort (HR: 7.02, 95% CI: 4.06–12.12, *p* < 0.001). These results also showed that age (HR: 1.02, 95% CI: 1.01–1.04, *p* = 0.010), tumor residual disease (HR: 1.34, 95% CI: 1.10–1.63, *p* = 0.004), and stage (HR: 1.61, 95% CI: 1.05–2.46, *p* = 0.027) served as independent prognostic risk factors (Table 2A). Multivariate Cox regression analysis was performed using the significant prognostic factors identified in the univariate analysis. The risk score model was further indicated to possess predictive performance ability for OS, owning the most significant prediction (HR: 5.24, 95% CI: 2.92–9.43, *p* < 0.001). Similar results were also obtained in the whole cohort, as the risk score model performed best in both univariate analysis (HR: 3.90, 95% CI: 2.61–5.81, *p* < 0.001) and multivariate analysis (HR: 3.01, 95% CI: 1.98–4.57, *p* < 0.001, Table 2B). Time–ROC and area under the curve (AUC) of each factor and all combined were also presented, and our risk score model showed a high AUC (Figures 5F–I). Pairwise Pearson’s correlation analysis was employed, and an adjusted *p* value was set up for a threshold to derive the regulation of genes enrolled in the risk score model and clinical pathological characteristics (Figure 5J). Besides CXCL14 and PI3, the other nine genes showed significant positive or negative correlations, indicating co-expression patterns between them. A significant positive relationship between venous invasion and lymphatic invasion was also observed, indicating an underlying cooperation between them in the long-term survival period in patients suffering from OV.

**TABLE 2:** Univariate **(A)** and multivariate **(B)** Cox regression analysis of the risk score model and other clinical variables.

Variables	Immune High cluster			Whole TCGA cohort		
	*N*	HR (95% CI)	*p* value	*N*	HR (95% CI)	*p* value
**A**						
Age	194	1.02 (1.01–1.04)	0.01	309	1.02 (1.01–1.04)	0.002
Stage	194	1.61 (1.05–2.46)	0.027	309	1.37 (0.98–1.92)	0.062
Grade	194	1.25 (0.67–2.32)	0.491	309	1.30 (0.83–2.02)	0.255
Lymphatic invasion	75	1.96 (0.66–5.85)	0.226	115	1.23 (0.63–2.42)	0.539
Tumor RD	173	1.34 (1.10–1.63)	0.004	278	1.33 (1.14–1.54)	<0.001
Venous invasion	54	0.75 (0.24–2.29)	0.611	84	0.69 (0.32–1.47)	0.334
Risk score model	194	7.02 (4.06–12.12)	<0.001	309	3.90 (2.61–5.81)	<0.001
**B**						
Risk score model	173	5.24 (2.92–9.43)	<0.001	278	3.01 (1.98–4.57)	<0.001
Age	173	1.02 (1.00–1.04)	0.11	278	1.02 (1.00–1.03)	0.02
Stage	173	1.52 (0.96–2.43)	0.08	278	1.28 (0.89–1.84)	0.18
Tumor RD	173	1.17 (0.94–1.47)	0.15	278	1.18 (1.01–1.39)	0.04

**FIGURE 6:**
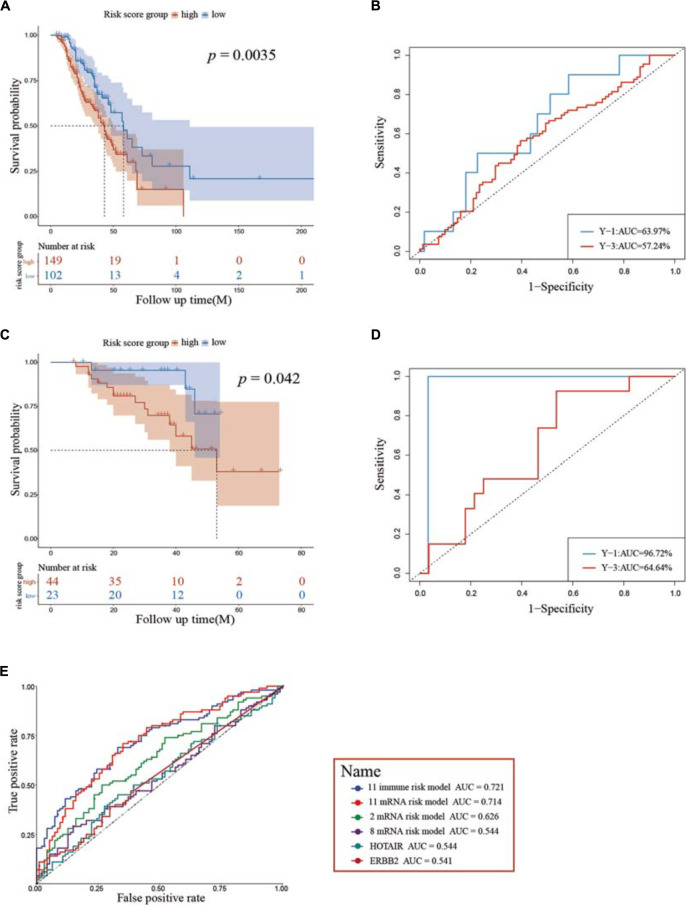
Assessment of prognostic capacity using the two external cohorts GSE9891 **(A,B)** and GSE14764 **(C,D)**, respectively. **(E)** ROC curves show the sensitivity and specificity of the immune risk model signature and other known biomarkers in predicting the OS of patients. ROC, receiver operating characteristic; AUC, area under the curve.

Two independent cohorts – GSE9891 and GSE14764 – were then employed as external validations for the model to confirm prognostic accuracy, and each was grouped into high and low groups using the same algorithm aforementioned. Kaplan–Meier curves showed significant differences in GSE9891 (log-rank test, *p* = 0.0035) and GSE14764 (log-rank test, *p* = 0.042), similar to our previous results, and time–ROC indicated that our immune risk score model had high sensitivity and specificity to predict survival probability (Figures 6A–D). Also, we verified our model to possess stable and reliable ability compared with several biomarkers identified previously (Figure 6E). Hence, these results indicated that our model is reliable in making a precise prediction.

### Stratification and Validation Among Clinical Pathological Factors as a Predicted Indicator

**FIGURE 7:**
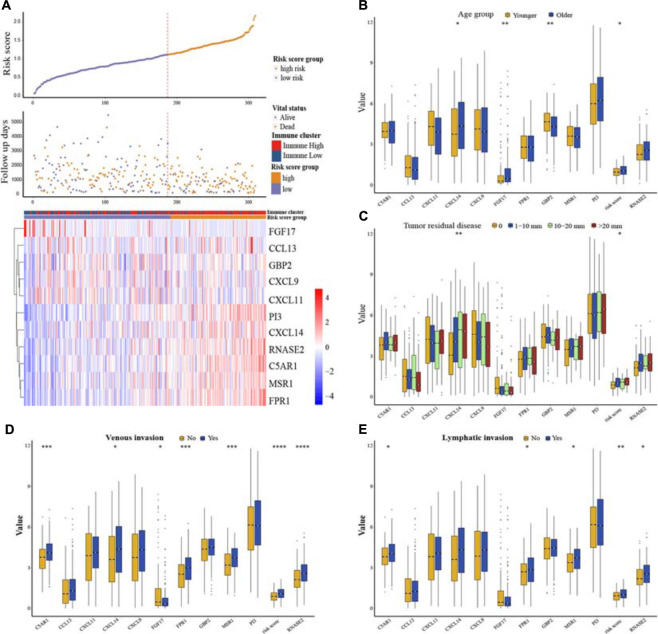
Risk score distribution and clinical stratification for the whole TCGA cohort. **(A)** The distribution of risk scores, survival status, and expression pattern of immune genes in the whole TCGA dataset. **(B)** OV patients were divided into two groups according to median age, and then the relationships among risk score distribution and expression levels of immune genes between age groups were explored. **(C)** Relationships among risk score distribution, expression levels of immune genes, and different status of tumor residual disease. **(D)** Relationships among risk score distribution, expression levels of immune genes, and different status of venous invasion. **(E)** Relationships among risk score distribution, expression levels of immune genes, and different status of lymphatic invasion. ^∗^*p* < 0.05, ^∗∗^*p* < 0.01, ^∗∗∗^*p* < 0.001, ^****^p < 0.0001.

We explored the distribution of risk scores and survival time in the whole TCGA cohort, annotated with the distribution of expression pattern of immune genes between two immune clusters (Figure 7A). Patients with higher risk scores tended to have shorter survival time and more likely remained in the “Immune High” cluster, whereas patients with lower risk scores tended to have longer survival time and also more likely remained in the “Immune Low” cluster. Moreover, we specifically explored the distribution of risk scores and expression levels of each gene in the whole cohort stratified by different clinical characteristics. As previously described, risk scores in the older group (median age > 58 years old) gained higher levels, accompanied by higher expression levels of CXCL14 and FGF17 and lower expression levels of GBP2 (Wilcoxon signed-rank test, *p* < 0.05, Figure 7B). Patients with increasing tumor residual disease diameters similarly gained higher values of risk scores (Kruskal–Wallis test, *p* < 0.05, Figure 7C). While the number of patients in the current study with either venous invasion or lymphatic invasion was comparatively small, we did observe some notable and significant intergroup differences among immune genes using the prognostic model, indicating its potential prognostic role in diverse pathological situations (Wilcoxon signed-rank test, *p* < 0.05, Figures 7D,E).

### Tumor Immune Landscape and Upstream Regulatory Mechanisms

**FIGURE 8:**
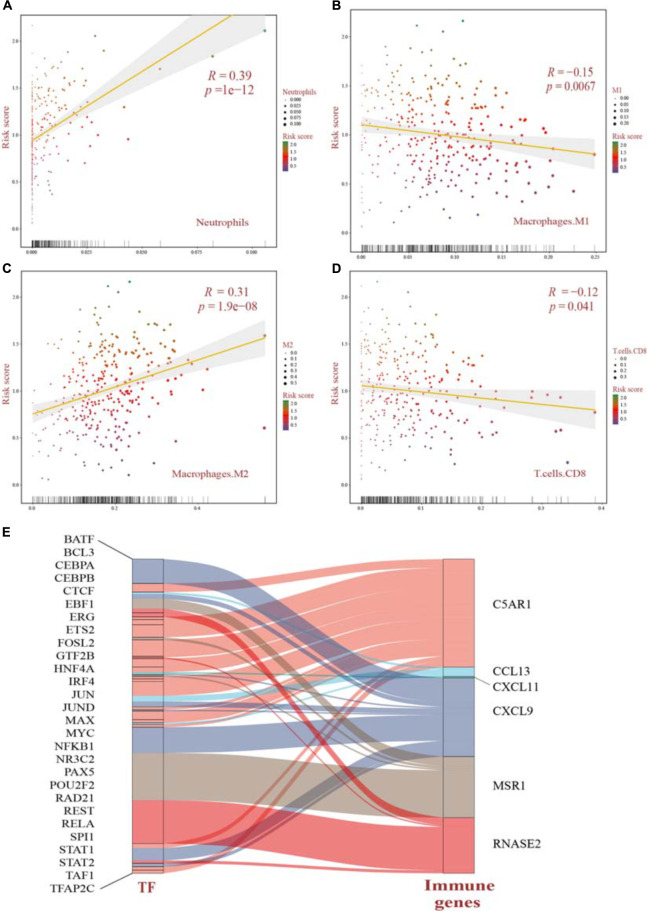
**(A–D)** Pearson’s correlation analysis of the risk score distribution and immune cell infiltration significantly identified by CIBERSORT in OV patients. All *p* values were corrected by the BH method. **(E)** Upstream regulatory mechanisms revealed the network among TFs and immune genes using the Sankey plot. The width of each alluvial stripe in the TF column represents -log2 (*p* values) of pairwise Pearson’s correlation adjusted. TFs, transcription factors.

Three hundred and nine samples were further quantified for a view of tumor immune landscape using the CIBERSORT method, as well as the different identification between the two clusters, which pointed out proportion changes in the immune microenvironment (Supplementary Figure 4). We inspected the association between risk score distribution and each of the 22 leukocyte cells. Pairwise Pearson’s correlation analysis indicated that the risk score model had an intimate relationship with immune component changes, suggesting diversities of systematic antitumor treatment on changes derived from cell-mediated immune response prospectively. Among neutrophils (*R* = 0.39), M2 macrophages (*R* = 0.31) were positively associated with risk score (*p* < 0.05), indicating a worse prognosis, while M1 macrophages (*R* =−0.15) and CD8 T cells (*R* =−0.12) presented negative associations, hence, a relative better prognosis (*p* < 0.05) (Figures 8A–D and Supplementary Figure 5). Additionally, we examined the upstream regulatory mechanisms of the genes contributing to the prognostic model. Transcription factor (TF) datasets were downloaded from TRRUST (version 2) (Han et al., 2018) and LncMAP databases (Li et al., 2018). Combined with the expression of all the TFs and immune-related genes, Pearson’s correlation analysis was applied and then a Sankey plot was shown after integrating a significant pairwise comparison of the TF–immune gene regulatory network (Figure 8E). A total of 29 TF–gene pairs targeting 6 immune genes significantly were extracted from 63 pairs (Supplementary Table 4). While CXCL11 targeted by JUN owned a negative regulatory mechanism (*R* = −0.142, adjusted *p* = 0.0293), all the other TFs were observed to have positive relationships while targeting immune genes, respectively.

## Discussion

Using TCGA dataset in an unbiased manner, we systematically evaluated the TME in OV by multiple approaches to investigate immune activity and proposed prognostic analyses based on bulk immune genes. A risk score model was then constructed and further analyzed by machine learning through different immune clusters and clinical pathological variables. Association with the model-stratified groups identified a significant correlation with OS, suggesting that it provided an independent and reliable measurement for gyneco-oncologists to ensure appropriate pre- or post-surgery treatment and chemotherapy management which is, to some extent, crucial and urgent, due to increasing incidence, lower 5-year survival rate, higher rate of recurrence, and resistance to chemotherapy in OV (Dao et al., 2016; Torre et al., 2018; Siegel et al., 2020).

The unsupervised hierarchical clustering analysis based on ssGSEA identified two clusters that presented obvious differences of immune activity and TME heterogeneity estimated by the ESTIMATE algorithm. Inverse correlations with tumor purity were seen for most expression of immune genes (Li et al., 2016; Rhee et al., 2018), as stromal cells and immune cells are the main non-tumor components co-existing with malignant cells in bulk tumor specimen, by which the tumor purity is deduced. These non-tumor cells dilute the purity of OV cells in TME. The cytolytic activity defined as the geometric mean of GZMA and PRF1 confirmed this robust stratification for the two clusters, for their ultimate effect mechanisms in the cancer immunity cycle. However, no significant difference in overall survival time was observed between these two different clusters, suggesting that genomic alterations may play dominant roles in affecting the functionality of immune cells *via* modification of TME and, finally, the immunotherapy response (Abdalla et al., 2014). Genomic expression data were further utilized for expanding the depth of understanding. We identified 381 DEGs, followed by GO and KEGG analyses, and also GSEA utilizing two gene sets as genetic backgrounds revealed that significantly enriched terms were observed in immune or inflammatory pathways, underlying their associations with immune activation or inhibition mechanisms in the progression of OV. For instance, it has been proven that high levels of IL-6, a major immunoregulatory cytokine, are associated with TME alterations by binding to its specific receptor IL-6R, whose increased expression, as well as its soluble spliced variant, is regulated by tumor-associated inflammation, leading to death (Rath et al., 2010; Lane et al., 2011). Epithelial–mesenchymal transition (EMT) is a well-known mechanism involved in the biological process of tumorigenesis and resistance to adjuvant therapies, such as paclitaxel-resistant OV cells (Kajiyama et al., 2007; Savagner, 2010). It shed light on blocking the EMT pathways for preventing tumor migration and invasion, at the same time remodeling to chemotherapy and immunity therapy (Rosano et al., 2011; Du et al., 2013). By activating NF-κB, OV cells overexpressed Her2/neu to induce the expression of VEGF which substantially increases vascular permeability, suggesting its involvement in the formation of malignant ascites (Hsieh et al., 2004). Previous studies have already approved the immune-related mechanisms in pan-cancer through TCGA and GEO databases (Charoentong et al., 2017) and the immunotherapeutic strategy available for multiple cancer types such as melanoma (Pandolfi et al., 2008; Achkar and Tarhini, 2017) and non-small cell lung cancer (Herbst et al., 2018), narrowly but successfully, except for OV as a result of low mutational burden or other reasons. In the current study, aiming to genotype and immunophenotype relations involved in OV, we filtered the expression of immune genes from the ImmPort database. LASSO Cox regression analysis identified MSR1, FPR1, RNASE2, GBP2, CXCL9, CXCL11, C5AR1, CCL13, FGF17, CXCL14, and PI3 as hub genes. Among these, FPR1 participates in tumorigenicity of human cervical cancer cells *via* activation of immune cells induced by N-formyl peptide (Cao and Zhang, 2018; Minopoli et al., 2019). CXCL9 and CXCL11 have been associated with activation of Th1 immunity within TME and a favorable response to chemotherapy and immunotherapy in melanoma (Harlin et al., 2009; Hong et al., 2011). CCL13 can be expressed by M2 macrophages with both anti-inflammatory and tissue repair functions (Grage-Griebenow et al., 2001; Murray and Wynn, 2011). It could be speculated that these biomarkers might play vital roles in the carcinogenesis of OV. Here, we chose the parameter family = “cox” as a response type to filter genes associated with OS. Moreover, considering smaller variables, we also applied other methods to narrow target genes based on AIC or multivariable Cox regression. However, these results failed to reach higher AUCs, suggesting an optimum balance employing genes. Then, a risk score model was constructed as a linear fit form and validated on testing sets as well as on two GEO datasets. Our results predominantly indicated that the risk score model could predict prognosis, as a higher score accompanies a worse prognosis. Besides, focusing on the whole TCGA cohort, a higher risk score may more likely incline to the “Immune High” cluster with a truncated survival time. With more genome-wide annotations’ acquisition, bipartite regulation, especially TF-regulating networks, is highly specific to different cell types (Neph et al., 2012). Upstream regulatory mechanisms were then explored and more molecular interactions were gained, which means a complex co-regulation network exists while considering the immune-related events in OV that need further exploration.

Univariate and multivariate Cox regression analyses were explored and identified independent clinical predictors additionally. As a result, the risk score model performed well entirely, indicating a reverse association with prognosis. Unexpectedly, age was screened out as an independent predictor in the whole TCGA cohort. Various changes occur during aging, while aging stimulates senescence *in vivo* (Boulestreau et al., 2020). Accompanying senescence, hematopoietic and immune health both decline significantly, contributing to an impaired immune function in the elderly (Beerman et al., 2010). However, previous studies often prove age as a non-significant predictor in OV or other cancers (Liu et al., 2020b). In the current study, we filtered out OS < 90 days for the purpose of long-term influence on OS and treatment management. Tumor residual disease (RD), that is, lesion diameters after cytoreductive surgery, is a consistently important factor across molecular subtypes and disease patterns (Aletti et al., 2007; Chi et al., 2009; Wallace et al., 2017; Wang et al., 2017). Minimizing RD remains an important goal to improve OS when feasible. A multivariate analysis controlling for age, preoperative albumin, stage, disease dissemination pattern, molecular subtypes, and RD posed that RD is the only variable independently associated with OS (Torres et al., 2018). Here, we confirmed the above statements. We observed a significant positive relationship between venous invasion and lymphatic invasion, suggesting their roles involved in immune regulation for a long-term survival incidence. Tumoral lymphovascular space invasion (LVSI), defined as the presence of tumor cells inside capillary lumens of either a lymphatic or a microvascular system within OV, has been reported as a new biomarker of progression (Matsuo et al., 2012, 2014). Moreover, we have set up and proved an enlightening and independent method for gyneco-oncologists and pathologists detecting immune-related genes to stratify prognosis of patients with OV.

Again, we employed CIBERSORT inferring quantitatively infiltrating lymphocytes from tumor transcriptomes. These results not only demonstrated diversity between immune clusters but also showed significant Pearson’s correlations between the risk score model and leukocytes. Previous literature shows that the complexity of the TME determines the functions of immune cells, especially those, such as neutrophils, with dual functions (Medina-Echeverz et al., 2014). Polarizing from M0 macrophages, the distinct immunoregulatory functions of activated M1 and M2 macrophages are antitumoral and protumoral, respectively (Noy and Pollard, 2014). Moreover, lymphocytes can strengthen cancer immune surveillance to suppress tumor cell proliferation, invasion, and metastasis (Dunn et al., 2004). The levels of tumor-infiltrated CD8+ T cells in the HGSOC tumors reveal a positive correlation with the patients’ survival regardless of the extent of residual disease, therapy, or BRCA1 mutation (Ovarian Tumor Tissue Analysis Consortium et al., 2017). Combining all the results mentioned above, we characterized the immune landscape with a risk score model and introduced a novel biomarker to predict prognosis which can further guide treatment decisions in patients with OV.

To conclude, our study has proposed multiple methods to investigate the TME landscape. Genes and the risk score model based on immune clusters were analyzed. We found that the risk score model was significantly associated with OV prognosis. Further analyses indicated that the risk score model was independent and more sensitive and specific than other clinical characteristics. Thus, we strongly believe that the immune-related model represents an important contribution and will enhance the identification of complex mechanistic insights of heterogeneity in OV.

## Data Availability Statement

Publicly available datasets were analyzed in this study. This data can be found here: https://xena.ucsc.edu/; https://www.gencodegenes.org/human/release_22.html; https://www.ncbi.nlm.nih.gov/geo/; and https://www.immport.org.

## Author Contributions

GZ designed and directed all the research. XZ, SC, QG, YC, TL, and JW performed the data processing and experimental analysis. GZ and XZ drafted the manuscript. All authors reviewed and approved the final version of the manuscript.

## Conflict of Interest

The authors declare that the research was conducted in the absence of any commercial or financial relationships that could be construed as a potential conflict of interest.

## Footnotes

1 https://xena.ucsc.edu/

2 https://www.gencodegenes.org/human/release_22.html

3 https://www.ncbi.nlm.nih.gov/geo/

4 https://www.immport.org

5 https://www.r-project.org/
