# Supplementary material for: Combination of Immune-Related Genomic Alterations Reveals Immune Characterization and Prediction of Different Prognostic Risks in Ovarian Cancer
Source: Front Cell Dev Biol. 2021 Apr 23;9:653357. doi: 10.3389/fcell.2021.653357 (PMC8102990; doi:10.3389/fcell.2021.653357)
Supplement: Supplementary file 1 [file Data_Sheet_1.PDF]

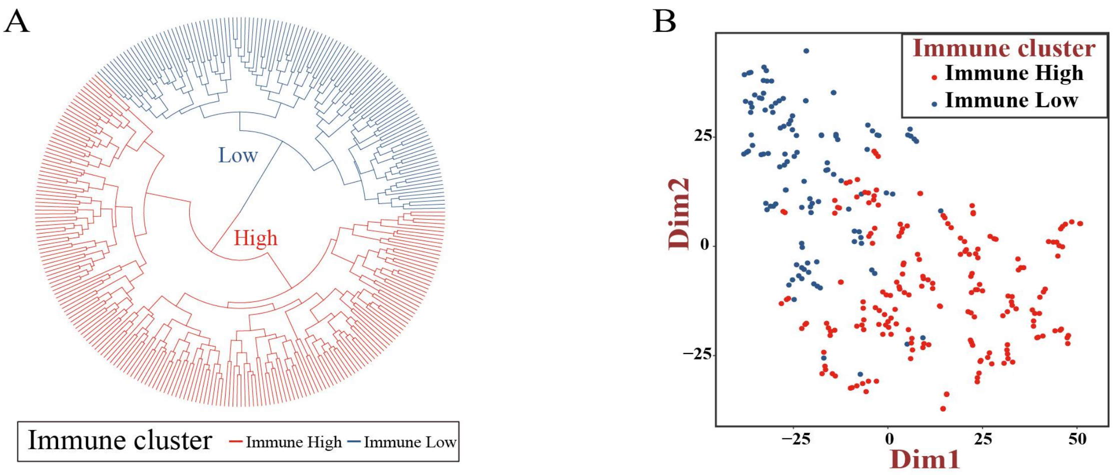

Supplementary Figure 1. (**A**) Unsupervised hierarchical clustering analysis showed an obvious difference of composition in TCGA OV. (**B**) t-SNE analysis supported this stratification of two immune-associated categories.
