# Supplementary material for: Combination of Immune-Related Genomic Alterations Reveals Immune Characterization and Prediction of Different Prognostic Risks in Ovarian Cancer
Source: Front Cell Dev Biol. 2021 Apr 23;9:653357. doi: 10.3389/fcell.2021.653357 (PMC8102990; doi:10.3389/fcell.2021.653357)
Supplement: Supplementary file 2 [file Data_Sheet_2.PDF]

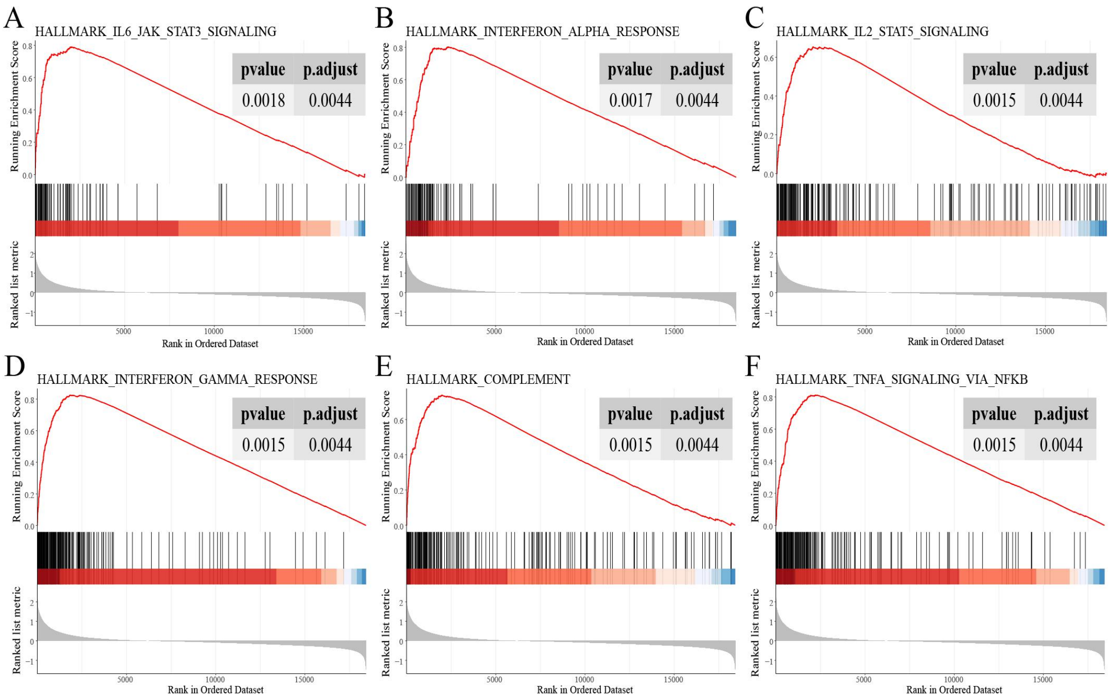

Supplementary Figure 2. Significant immune enriched terms in "hallmarker gene sets" for GSEA. (**A**) IL6_JAK_STAT3_SIGNALING, (**B**) INTERFERON_ALPHA_RESPONSE, (**C**) IL2_STAT5_SIGNALING, (**D**)INTERFERON_GAMMA_RESPONSE, (**E**) COMPLEMENT, (**F**) TNFA_SIGNALING_VIA_NFKB. All adjusted *p* values<0.05 were seen as statistical threshold.
