# Supplementary material for: Combination of Immune-Related Genomic Alterations Reveals Immune Characterization and Prediction of Different Prognostic Risks in Ovarian Cancer
Source: Front Cell Dev Biol. 2021 Apr 23;9:653357. doi: 10.3389/fcell.2021.653357 (PMC8102990; doi:10.3389/fcell.2021.653357)
Supplement: Supplementary file 3 [file Data_Sheet_3.PDF]

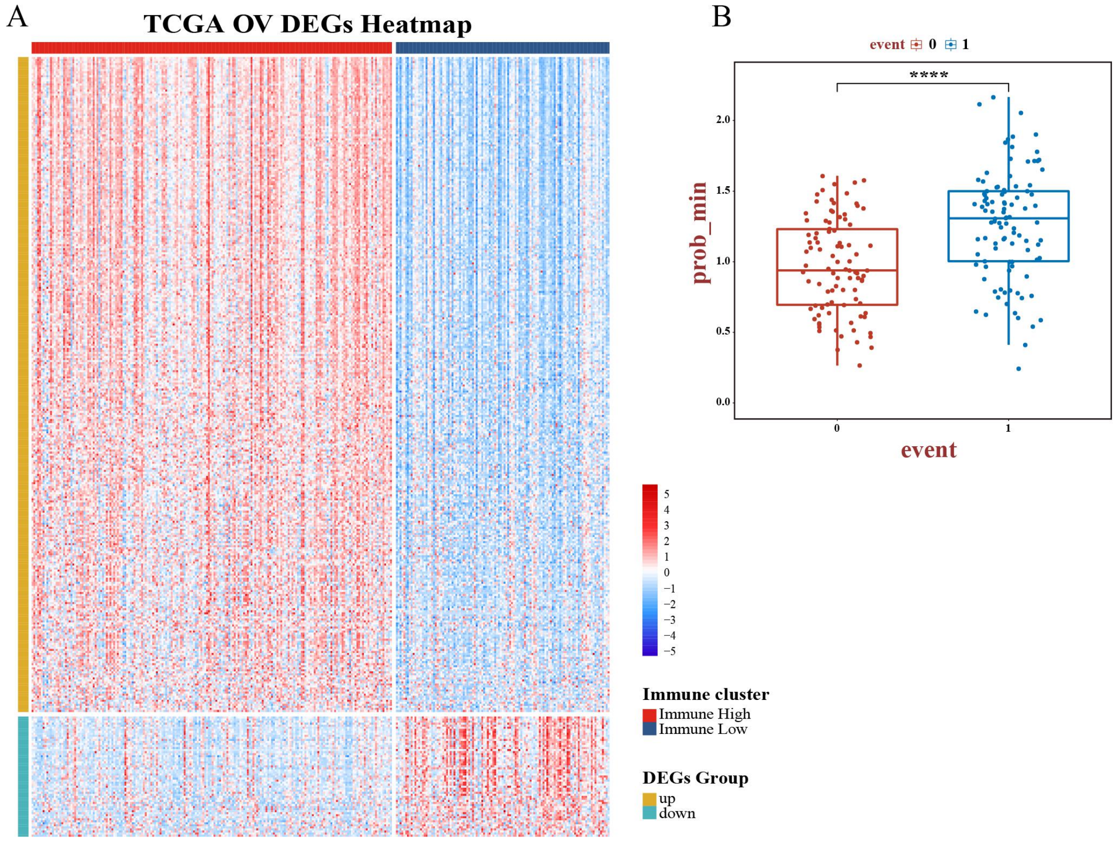

Supplementary Figure 3. (**A**) Landscape of expression pattern for all DEGs between two immune clusters. (**B**) Risk score model derived from LASSO Cox regression analysis performed precisely statistically for predicting OS in training set. 0 means alive, while 1 means death. DEGs, differentially expressed genes; ****p<0.0001.
