# Supplementary material for: Combination of Immune-Related Genomic Alterations Reveals Immune Characterization and Prediction of Different Prognostic Risks in Ovarian Cancer
Source: Front Cell Dev Biol. 2021 Apr 23;9:653357. doi: 10.3389/fcell.2021.653357 (PMC8102990; doi:10.3389/fcell.2021.653357)
Supplement: Supplementary file 4 [file Data_Sheet_4.PDF]

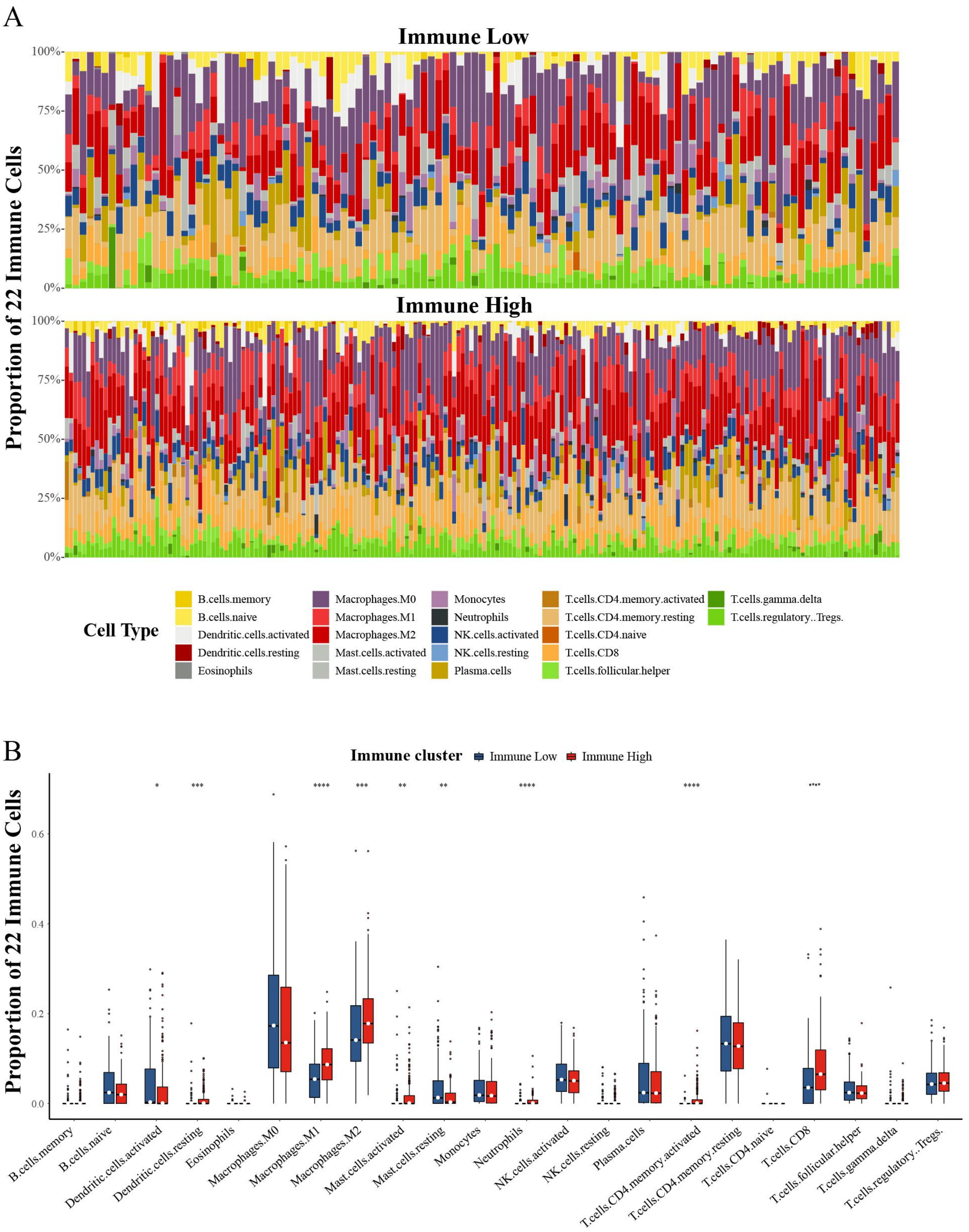

Supplementary Figure 4. (**A**) A landscape of LM22 in 309 samples quantified using CIBERSORT method. (**B**) Different identification of LM22 between two immune clusters. **p*<0.05, ***p*<0.01, ****p*<0.001, *****p*<0.0001.
