# Supplementary material for: Combination of Immune-Related Genomic Alterations Reveals Immune Characterization and Prediction of Different Prognostic Risks in Ovarian Cancer
Source: Front Cell Dev Biol. 2021 Apr 23;9:653357. doi: 10.3389/fcell.2021.653357 (PMC8102990; doi:10.3389/fcell.2021.653357)
Supplement: Supplementary file 5 [file Data_Sheet_5.PDF]

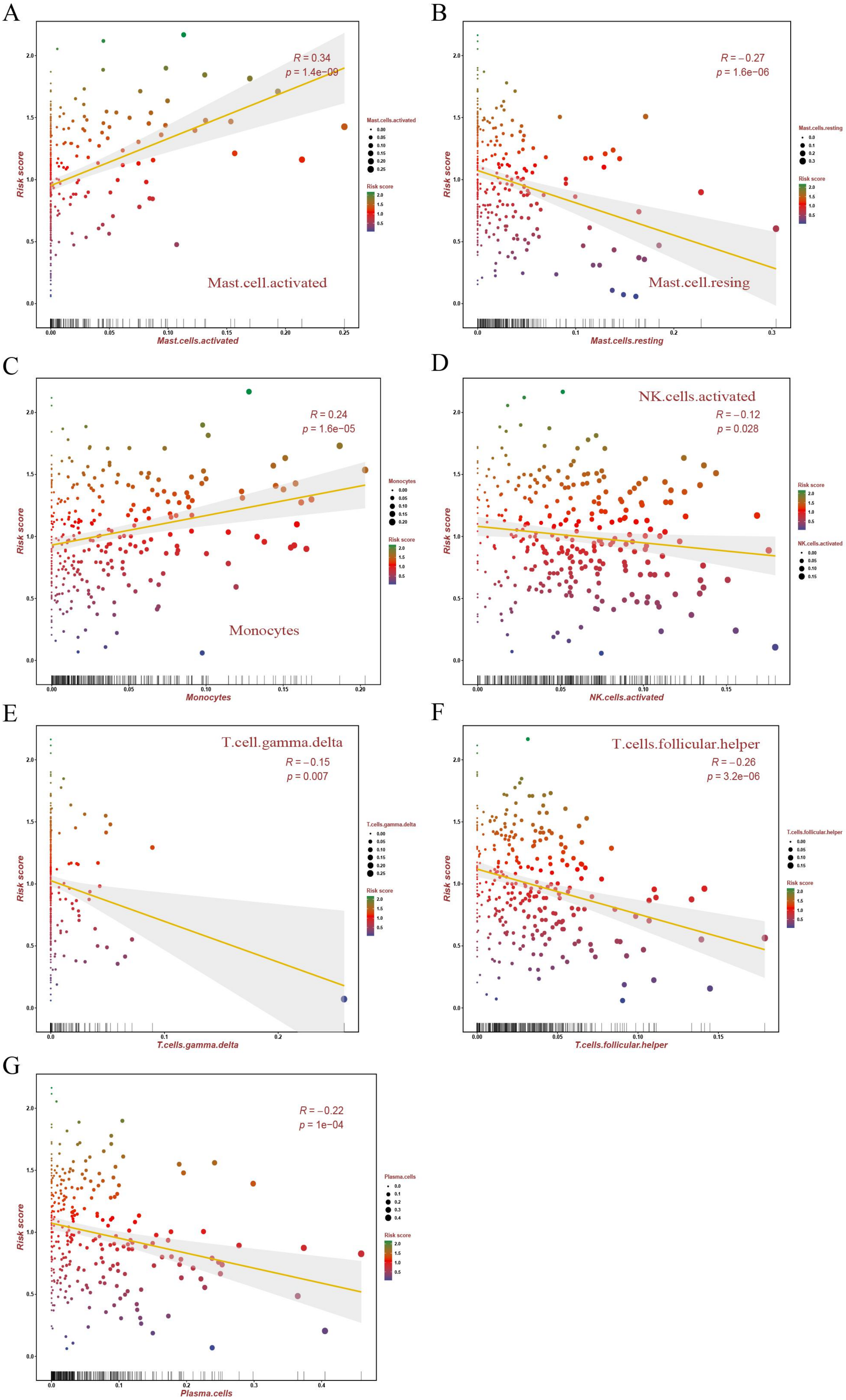
A
R = 0.34
p = 1.4e−09
Mast.cell.activated
Mast.cells.activated
Risk score
B
R = −0.27
p = 1.6e−06
Mast.cell.resing
Mast.cells.resting
Risk score
C
R = 0.24
p = 1.6e−05
Monocytes
Risk score
D
NK.cells.activated
R = −0.12
p = 0.028
Risk score
E
T.cell.gamma.delta
R = −0.15
p = 0.007
T.cells.gamma.delta
Risk score
F
T.cells.follicular.helper
R = −0.26
p = 3.2e−06
Risk score
G
R = −0.22
p = 1e−04
Plasma.cells
Risk score

Supplementary Figure 5. Pearson's correlation analysis of the risk score distribution and immune cell infiltration significantly identified by CIBERSORT in OV patients. All *p* values were corrected by BH method.
